# Transjugular antegrade transvenous obliteration, with and without portal decompression, for management of rectal variceal hemorrhage

**DOI:** 10.1186/s42155-024-00479-x

**Published:** 2024-09-04

**Authors:** Gabriel E. Li, Jeffrey Forris Beecham Chick, Eric J. Monroe, Matthew Abad-Santos, Ethan W. Hua, David S. Shin

**Affiliations:** 1 Division of Interventional Radiology, Department of Radiology, University of Washington, 1959 Northeast Pacific Street, Seattle, WA 98195 USA; 2 The Deep Vein Institute, University of Washington, 1959 Northeast Pacific Street, Seattle, WA 98195 USA; 3 Section of Vascular and Interventional Radiology, Department of Radiology, University of Wisconsin, 600 Highland Avenue, Madison, WI 53792 USA; 4 Division of Vascular and Interventional, Radiology Department of Radiology, University of Southern California, 1500 San Pablo St Second Floor Imaging, Los Angeles, CA 90033 USA

**Keywords:** Rectal varices, Rectal hemorrhage, Balloon-occluded antegrade transvenous obliteration, BATO, Sclerosis, Transjugular intrahepatic portosystemic shunt creation, TIPS, Portal decompression

## Abstract

**Purpose:**

To report antegrade transvenous obliteration, with or without concurrent portosystemic shunt creation, for the treatment of hemorrhagic rectal varices.

**Materials and methods:**

Eight patients, including five (62.5%) females and three (37.5%) males, with mean age of 55.8 ± 13.8 years (range: 30–70 years), underwent transjugular-approach antegrade transvenous obliteration of rectal varices, with or without portosystemic shunt creation. Demographic data, procedural details, technical success of variceal obliteration, clinical success, adverse events, and follow-up outcomes were retrospectively recorded. Clinical success was defined as resolution of rectal hemorrhage.

**Results:**

Portal venous access was achieved via a transjugular intrahepatic approach in all patients. The inferior mesenteric vein was selected, and foamy sclerosant (1:2:3 mixture by volume of ethiodized oil: sodium tetradecyl sulfate: air) was injected into the rectal varices with antegrade balloon occlusion in seven (87.5%) and without balloon occlusion in one (12.5%). Five of eight (62.5%) patients underwent concomitant transjugular intrahepatic portosystemic shunt (TIPS) creation (mean diameter 8.4 ± 0.9-mm) immediately following transvenous obliteration. Technical success of variceal obliteration was achieved in all patients. There were no immediate post-procedural adverse events. There were no reported occurrences of rectal ischemia, perforation, or stricture following obliteration. Two (40%) of the patients who underwent concomitant TIPS creation developed hepatic encephalopathy within 30 days of the procedure, which was medically managed. Clinical resolution of hemorrhage was achieved in all patients with no recurrent rectal variceal hemorrhage during mean follow-up of 666 ± 396 days (range: 14 − 1,224 days).

**Conclusion:**

Transvenous obliteration, with or without concurrent TIPS creation, is feasible with promising results for the management of rectal variceal hemorrhage.

## Introduction

Anorectal ectopic varices are under-recognized in patients with portal hypertension [1]. While hemorrhage from these varices is uncommon, it may be life-threatening and difficult to manage [2]. Treatment options include medical management, endoscopic sclerotherapy or ligation, endovascular transvenous obliteration, transjugular intrahepatic portosystemic shunt (TIPS) creation, and surgical ligation [3–6]. Data on endovascular interventions for rectal varices are limited to case reports and series [7–9]. There are no consensus treatment algorithms, and management strategies vary widely.

The purpose of this study is to report the efficacy and safety of transjugular-approach antegrade transvenous obliteration, with or without portal decompression, for the management of rectal variceal hemorrhage.

## Materials and methods

### Study design, inclusion, and exclusion criteria

This single-center, retrospective study was conducted under *Institutional Review Board* approval and complied with the *Health Insurance Portability and Accountability Act*. All patients who underwent endovascular intervention for rectal variceal hemorrhage were identified (*n* = 13). Five (38.5%) patients were excluded, including four (30.8%) patients who were treated with TIPS creation alone and one (7.7%) patient who underwent variceal embolization without sclerosis.

### Patient demographics and presenting symptoms

Eight patients, including five (62.5%) females and three (37.5%) males, constituted the study cohort. All patients had cirrhotic portal hypertension. *Demographic data*,* including etiologies for cirrhosis*,* are shown in* Table 1. *Model for End-Stage Liver Disease (MELD) scores*,* for risk stratification*,* are shown in* Table 2. Rectal varices were diagnosed using endoscopy in five (62.5%) patients and contrast-enhanced computed tomography in three (37.5%) patients. All patients presented with bleeding per rectum leading to hemodynamic instability.

**Table 1:** Etiologies for cirrhosis

Etiology for Cirrhosis	*N* (%)
Alcohol	4 (50)
Cryptogenic	2 (25)
Non-alcoholic Steatohepatitis	1 (12.5)
Alcohol and Hepatitis C	1 (12.5)

**Table 2:** MELD scores by subgroup

Groups (*N*)	Mean ± Standard Deviation (Range)
All Patients (8)	19.25 ± 9.4 (7–33)
Transvenous Obliteration Only (3)	30.0 ± 3.0 (27–33)
Transvenous Obliteration + TIPS (5)	12.8 ± 3.6 (7–16)
Variceal Hemorrhage Treatment as Inpatient (6)	20.8 ± 10.6 (7–33)
Rebleeding Prophylaxis as Outpatient (2)	14.5 ± 0.7 (14–15)

Six (75%) patients were admitted with acute hemorrhage refractory to medical management. Two (25%) patients were stabilized and referred for elective endovascular treatment. All patients required blood transfusions during the index admission (mean 9.6 ± 9.6 units of packed red blood cells; range: 1–28 units). Previous failed treatments included attempted endoscopic management in three (37.5%) patients, attempted TIPS creation at an outside hospital in one (12.5%), and attempted surgical ligation in one (12.5%). Five (62.5%) patients had a surgical consultation prior to referral to interventional radiology and were deemed not to be surgical candidates.

### Variables and definitions

Demographic data, procedural details, technical success of variceal obliteration, clinical success, adverse events, and follow-up outcomes were retrospectively recorded.

Technical success was defined as administration of sclerosant into target rectal varices resulting in obliteration. Adverse events were categorized according to the *Adverse Event Classification by the Society of Interventional Radiology Standards of Practice Committee* [10]. Clinical success was defined as resolution of rectal hemorrhage.

### Procedure technique

**Fig. 1:**
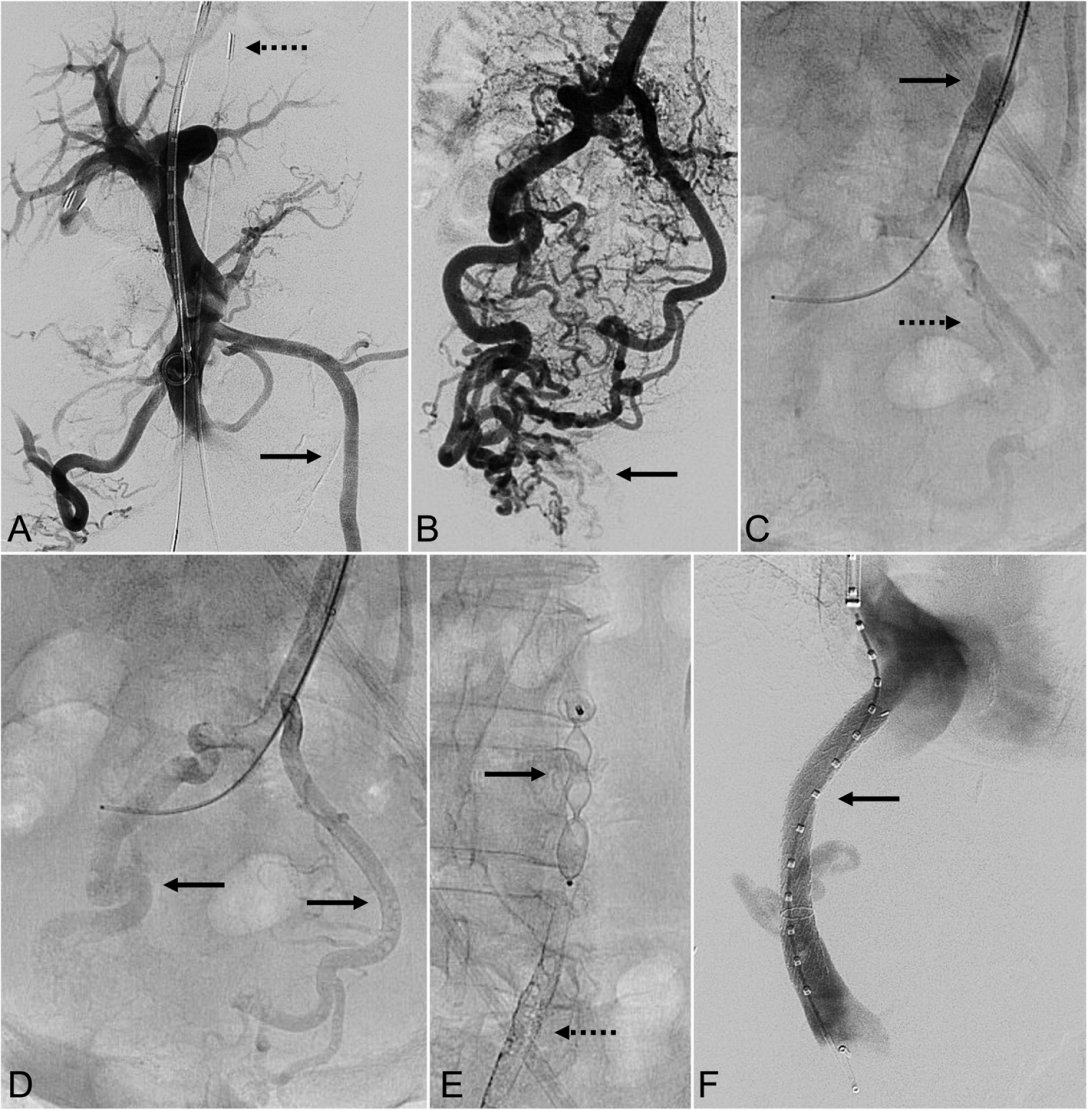
A *70-year-old female with bleeding rectal varices in the setting of non-alcoholic steatohepatitis cirrhosis. ***A **Digital subtraction portal venography following intravascular ultrasound-guided (dashed arrow) transjugular intrahepatic portal access, demonstrating retrograde flow down the enlarged inferior mesenteric vein (solid arrow). **B** Selective inferior mesenteric venography demonstrating extensive rectal varices (solid arrow). **C **Fluoroscopic image during sclerosant instillation with an occlusion balloon (solid arrow) inflated at the low inferior mesenteric vein trunk demonstrating sclerosant injection into the left superior rectal vein (dashed arrow). **D **Fluoroscopic image following further instillation demonstrating sclerosant in the bilateral superior rectal veins (solid arrows) and the rectal varices. **E** Fluoroscopic image demonstrating a 14 mm Amplatzer Vascular Plug II in the inferior mesenteric vein trunk (solid arrow) with the sclerosed vessels (dashed arrow). **F** Completion portal venography following transjugular intrahepatic portosystemic shunt placement with a 8–10 mm x 7/2 cm Viatorr TIPS stent dilated to 8 mm (solid arrow)

**Fig. 2:**
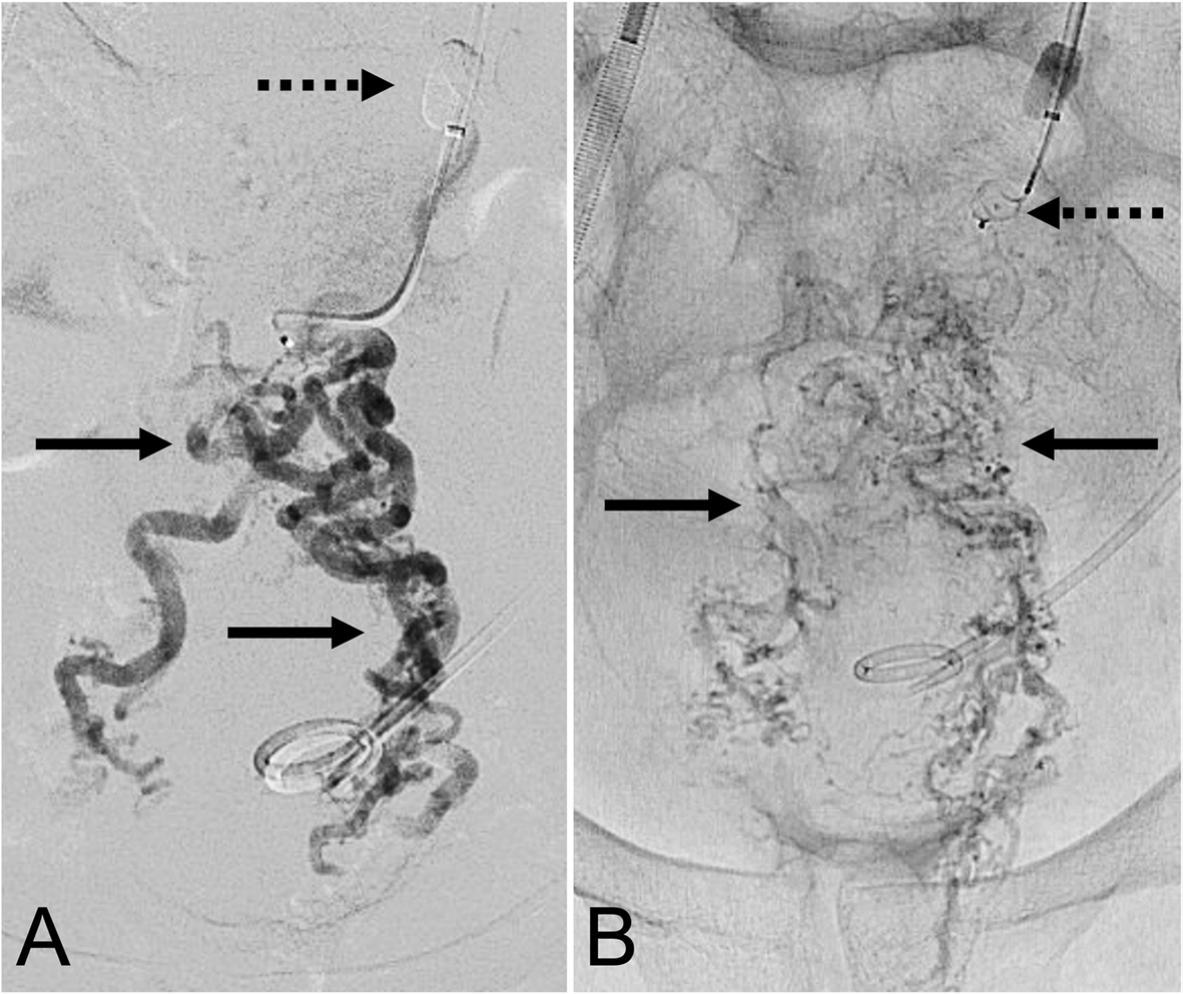
A *63-year-old female with bleeding rectal varices in the setting of liver failure. ***A** Selective inferior mesenteric venography demonstrating extensive rectal varices (solid arrows) with an occlusion balloon inflated (dashed arrow). **B** Fluoroscopic image following sclerosant instillation demonstrating sclerosant in the bilateral superior rectal veins and the rectal varices (solid arrows). An 8 mm Amplatzer Vascular Plug IV was placed in the inferior mesenteric vein trunk (dashed arrow). No TIPS was placed

*Procedures are detailed in* Figs. 1 and 2. Balloon-occluded antegrade transvenous obliteration (BATO) has previously been described [11–14]. All procedures were performed under general anesthesia (institutional preference). After portal access using intravascular ultrasound guidance, the inferior mesenteric vein was catheterized and venography was performed [15, 16]. An 8/9-French occlusion balloon catheter (Merci Device; Concentric Medical; Mountain View, CA) was then inflated in the inferior mesenteric vein to avoid retrograde flow during obliteration. Sclerosant mixture containing ethiodized oil (Lipiodol; Guerbet; Villepinte, France), 1% or 3% sodium tetradecyl sulfate (STS) (Sotradecol; Viatris; Canonsburg, PA), and air was then directly injected into the superior rectal veins and rectal varices using fluoroscopy. After the varices were obliterated, the confluence of the superior rectal veins and inferior mesenteric vein were embolized using one or more Amplatzer Vascular Plug II (Abbott Cardiovascular Systems; Abbott Park, IL). Further embolization using vascular coils was performed, as needed.

TIPS procedure occurred after obliteration as previously described [17–19]. Briefly, serial stent-graft (Gore Viatorr TIPS Endoprosthesis; Gore Medical; Newark, DE) dilation was performed until the portosystemic gradient was < 12 mmHg [20]. The decision whether or not to place a concurrent TIPS was made on a case-by-case basis from multidisciplinary discussion and operator discretion.

## Results

### Procedural details

Seven of eight (87.5%) patients underwent balloon occlusion-mediated obliteration. Balloon occlusion was not employed in one case due to operator preference. The sclerosant was mixed in varying ratios, but the most common was 1:2:3 of ethiodized oil:1% STS: air. Sclerosant volume was reported in two (25%) patients and was 15 mL in both. After obliteration, all patients underwent embolization of the inferior mesenteric vein with one-to-two Amplatzer Vascular Plug II with a mean diameter of 10.2 ± 2.6-mm (range: 8-1- mm). One (12.5%) patient underwent embolization of the bilateral superior rectal veins with multiple 5-6-mm coils. In five (62.5%) patients receiving concurrent TIPS creation, mean Viatorr stent-graft diameter was 8.4 ± 0.9-mm (range: 8-10-mm).

## Outcomes

Clinical success was achieved in all patients. One (12.5%) patient had concurrent external hemorrhoidal bleeding that required endoscopic treatment during the same hospitalization. Two (25%) patients, both in the obliteration alone cohort, underwent liver transplants 17 and 19 days after obliteration. No patients had any documented recurrence of rectal variceal bleeding during the length of the study.

In patients who received concurrent TIPS, the portosystemic gradient decreased from an initial mean gradient of 20.0 ​​± 8.6-mmHg to a final mean gradient of 8.2 ± 1.1-mmHg. MELD scores were lower in patients with concurrent TIPS than in the patients who had transvenous obliteration alone (12.8 ± 3.6 vs. 30.0 ± 3.0; *p* = 0.0004).

Mean length of follow up for patients in the study was 666 ± 396 days (range: 14 − 1,224 days). One (12.5%) patient, in the TIPS cohort, was lost to follow-up after 380 days.

### Adverse events

There were no immediate post-procedural adverse events. There were no reported occurrences of rectal ischemia, perforation, or stricture following the procedure. Two (40%) patients in the TIPS cohort developed hepatic encephalopathy that was managed medically without any TIPS revision.

Two (25%) patients died, 305 and 852 days after the procedure. One (12.5%) of these patients had concurrent TIPS placement. Neither death was due to variceal hemorrhage.

## Discussion

Rectal variceal hemorrhage in cirrhotic patients may be life-threatening and difficult to control. There are no validated algorithms or established guidelines for the management of this entity [2]. The present study supports the feasibility and efficacy of transvenous obliteration, with or without TIPS creation, for controlling rectal variceal bleeding. In this series, no recurrent rectal variceal hemorrhage or anorectal complications occurred during a mean follow-up period of 666 days.

Endoscopic therapy has been considered the first-line treatment for rectal variceal hemorrhage [2, 21] despite its low reported rates of durable clinical success. In one retrospective analysis, one-year recurrence rates for rectal variceal bleeding were 33.3% and 55.6% for endoscopic sclerotherapy injection and endoscopic band ligation, respectively [22]. Another study reporting the use of TIPS creation for ectopic varices noted that in twelve patients with rectal varices, five (41.7%) presented after prior treatment with endoscopic band ligation [23]. Surgical treatment is used as a salvage therapy for intractable bleeding [2, 21]. The most effective method is circumferential stapling, with a case series demonstrating successful control of rectal bleeding in nine of nine patients over a mean follow-up of 24 months [24]. However, many of the critically ill patients with hepatic decompensation are poor surgical candidates.

Endovascular treatment strategies include variceal embolization (e.g. using coils, plugs, or gelfoam), TIPS creation, and antegrade transvenous obliteration. Embolization alone is rarely performed and there is little data in the literature to evaluate its efficacy. Monotherapy with TIPS creation may be effective, with one series describing successful shunt placement in eleven patients with recurrent rectal variceal hemorrhage in one (9.1%) [23]. Embolization and TIPS placement, in concert, have been proposed as a more effective treatment option than either as monotherapy [7, 25]. Individual case studies have also demonstrated success of BRTO [26, 27], though this is often not technically feasible in rectal varices due to the absence of a catheterizable shunt outflow [28].

Prior case reports have found BATO effective in controlling rectal variceal bleeding [12, 14, 29]. However, none of the patients in these reports underwent concurrent TIPS placement due to contraindications. The present series included patients who underwent combination transvenous obliteration and TIPS creation (62.5%) and transvenous obliteration alone (37.5%), with both groups achieving durable hemostasis. As expected, the transvenous obliteration alone group had a significantly higher MELD score (30 vs. 13, *p* = 0.0004), leading to poor candidacy for TIPS creation. However, this group still benefited from the transjugular-approach technique with respect to the rectal variceal treatment.

Limitations of this study include its retrospective single-arm design and small sample size limiting ability to determine statistical significance and generalizability.

## Conclusion

Transjugular-approach transvenous obliteration, with or without concurrent TIPS creation, was feasible and resulted in control of rectal variceal hemorrhage.

## Data Availability

Data for this study available upon reasonable request.

## Abbreviations

TIPS: transjugular intrahepatic portosystemic shunt
MELD: Model for End-Stage Liver Disease
BATO: balloon-occluded antegrade transvenous obliteration
STS: sodium tetradecyl sulfate
